# Transient Hemolytic Anemia after Transjugular Intrahepatic Portosystemic Stent Shunt

**DOI:** 10.1155/1996/19343

**Published:** 1996

**Authors:** Sagrario Garcia-Rebollo, Emilio González-Reimers, Francisco Santolaria-Fernández, Francisco Diaz-Romero, Fermin Rodriguez-Moreno, Antonio Martinez-Riera

**Affiliations:** 1 Dptos. de Medicina Interna Hospital Universitario de Canarias La Laguna. Tenerife Canary Islands Spain; 2 Dpto de Y Radiologia Hospital Universitario de Canarias La Laguna. Tenerife Canary Islands Spain

## Abstract

Management of variceal bleeding secondary to portal hypertension constitutes a challenging issue, particularly in child's C cirrhotic patients. Recently, transjugular placement of self-expanding metallic stents in the liver (TIPS), creating a shunt between the portal and hepatic branches has provided a safe and promising therapeutic approach in this clinical situation. We report here the case of a 66-year-old male cirrhotic patient who developed a moderately severe clinical picture of a Coombsnegative hemolytic anemia (serum hemoglobin, 93 g/l, serum bilirubin 160.74 umol/L (9.4 mg/dl), indirect 6.3 mg/dl (107.73 umol/L); serum LDH 1220 u/l, reticulocytes, 5.1%. serum ferritin, 1221 ug/1, schistocytes in peripheral blood smear) the week after undergoing a TIPS, suggesting the development ofa microangiopathic hemolytic anaemia secondary to red blood cell disruption by passing through the metallic network of the stent.


*HPB Surgery*, 1996, Vol.9, pp.249–251
Reprints available directly from the publisher
Photocopying permitted by license only

© 1996 OPA (Overseas Publishers Association)
Amsterdam B.V. Published in The Netherlands
by Harwood Academic Publishers GmbH
Printed in Malaysia

### CASE REPORT

### Transient Hemolytic Anemia after Transjugular Intrahepatic Portosystemic Stent Shunt

SAGRARIO GARCIA-REBOLLO,* EMILIO GONZÁLEZ-REIMERS,* FRANCISCO SANTOLARIA-FERNÁNDEZ,* FRANCISCO DIAZ-ROMERO, ($) FERMIN RODRIGUEZ-MORENO* and ANTONIO MARTINEZ-RIERA

Dptos. de Medicina Interna (*) Y Radiologia ($). Hospital Universitario de Canarias. La Laguna. Tenerife. Canary Islands (Spain)

*(Received 12 March 1994)*

Management of variceal bleeding secondary to portal hypertension constitutes a challenging issue, particularly in child's C cirrhotic patients. Recently, transjugular placement of self-expanding metallic stents in the liver (TIPS), creating a shunt between the portal and hepatic branches has provided a safe and promising therapeutic approach in this clinical situation. We report here the case of a 66-year-old male cirrhotic patient who developed a moderately severe clinical picture of a Coombsnegative hemolytic anemia (serum hemoglobin, 93 g/1, serum bilirubin 160.74 umol/L (9.4 mg/dl), indirect 6.3 mg/dl (107.73 umol/L); serum LDH 1220 u/1, reticulocytes, 5.1%. serum ferritin, 1221 ug/l, schistocytes in peripheral blood smear) the week after undergoing a TIPS, suggesting the development of a microangiopathic hemolytic anaemia secondary to red blood cell disruption by passing through the metallic network of the stent.

KEY WORDS: TIPS-Portosystemic Shunt-Portal Hypertension-Cirrhosis

### INTRODUCTION

Variceal bleeding is a major complication of cirrhotic patients, associated with high mortality rates. Several therapeutic approaches are currently available, including pharmacological management with somatostatin and /or vasopressin, balloon tamponade, sclerotherapy, embolization, and porto systemic shunt [1], this last being associated with the lowest rate of rebleeding [2]. However, emergency shunt operations are usually reserved for patients in whom other therapies have been ineffective, mortality rates reaching figures as high as 50% among Child's C cirrhotics who undergo this intervention [3]. In recent times, self-expanding metallic stents have been placed in the liver, creating a shunt between the portal and hepatic branches (transjugular intrahepatic portosystemic shunt, TIPS), thus providing a promising approach for these clinical situations [4–9]. We report here the case of a patient who developed a moderately severe, transient clinical picture of hemolytic anemia after undergoing a TIPS, a complication not reported before, except perhaps for another case in which persistent hemolysis and encephalopathy developed [10].

### CASE REPORT

A 66-years-old male patient was referred to our hospital in order to undergo a TIPS. He had been treated in another center and liver cirrhosis had been diagnosed. He denied alcoholic intake, and there was a history of past blood transfusions in the course of a theracoplasty. Antibodies to hepatitis C virus were positive. In the last 4 months he had presented with eight episodes of variceal bleeding, treated with

*Correspondence to:* Emilio González-Reimers Dpto. de Medicina Interna. Hospital Universitario de Canarias. La Laguna. Tenerife. Canary Islands (Spain).

sclerotherapy and/or balloon tamponade together with pharmacological measures and blood transfusions. During his stay in that hospital, serum bilirubin, although slightly elevated at admission (42.75 umol/l), dropped to normal values afterwards. Prothrombin activity was 50%, and the patient developed ascites. A new bleeding episode due to variceal rupture, 24 hours after a sclerotherapy session lead his doctors to send him to our hospital.

At admission to our hospital a TIPS was performed, placing a 46 mm long metallic stent between the right hepatic vein and the portal system. However, this procedure neither caused a normalization of portal pressure, nor stopped variceal bleeding-the patient received another transfusion-, so another stent, 75 mm lenght, was inserted 5 days later, using techniques already described [6].

Bleeding immediately stopped after placement of this second device, portal pressure dropping from 36 to 17 cm $H_2O$. In the following days, however, serum bilirubin progressively increased (from 58.14 umol/L to 160.74 umol/L), indirect (107.73 umol/L) (Fig. 1), hemoglobin fluctuating between 90 and 91 g/l. This elevation in serum bilirubin was accompanied by an elevation in serum LDH (fig 1), reaching maximum levels of 1210 U/l; Coombs test was negative, serum ferritin reached 1221 ug/l, and haptogolobin was undetectable. Schistocytes were observed in the peripheral blood smear. Reticulocytes increased markedly, (153.000/mm3, 5.1%). A week later, bilirubin dropped (71.82 umol/L (4.2 mg/dl), indirect 35.96 umol/L (2.1 md/dl)) and later the, jaundice disappeared, total bilirubin dropping to 42.75 umol/L, serum LDH, to 645 U/l, and hemoglobin raising to 124 g/l. Ascites also disappeared and the patient did not present any sign of encephalopathy. No rebleeding has been observed.

### DISCUSSION

Transjugular installation of intrahepatic self-expanding metallic stents seems to constitute an excellent alternative to surgical portocaval shunts. Although some major complications-including death-have been described [8, 10], major problems are thrombosis and stenosis of the artificially-created shunts, although followup portography and radilation by further angioplasty may prevent these complications [7]. Indeed, after placement of the metallic device a neointima gradually develops, and sometimes it contributes to stenosis and thrombosis of the stent. However, before the intima grows, blood cells are forced through the metallic network, the possibility existing of red blood cell rupture leading to variable degrees of microangiopathic hemolytic anemia. We believe that our patient developed such a clinical picture. Although he

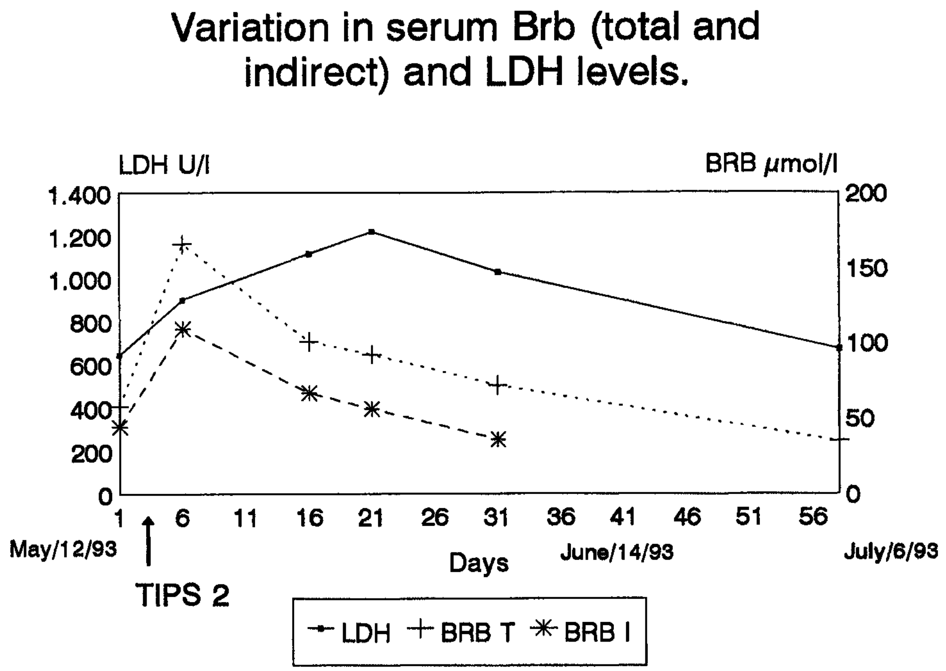

Figure 1 Variation in serum bilirubin (BRBT=total bilirubin; BRBI = indirect bilirubin) and LDH levels.

received transfusions during the stay in the other hospital, serum bilirubin was normal at that time; although he also received a transfusion before the second TIPS was performed, the rise in serum bilirubin was marked and reached its maximum not in the first days, but one week later. Recovery of this situation occurred spontaneously, patient is asymptomatic, without jaundice and with normal bilirubin values. Perhaps, recovery of hemolysis is concomitant with the development of a neointima which partially covers even the free portions of the metallic network and therefore diminishes red blood cell rupture. In another case described, hemolysis was persistent, only subsiding after liver transplantation and removal of the Wallstent [10]; it was supposed that blood flow through the wire mesh of the free portion of the stent caused intravascular hemolysis.

Thus, our case illustrates a rarely described but expectable complication of TIPS, i.e., a transient microangiopathic hemolytic anemia probably due to red blood cell rupture by passing through the metallic network of the intrahepatic stent.

### REFERENCES

1. Cello J.P, Grendell J.H, Crass R.A, Weber T.E, Trunkey DD. (1987) Endoscopic sclerotherapy versus portocaval shunt in patients with severe cirrhosis and acute variceal hemorrhage. *N Eng J Med*, **316**: 11–15.
2. Reynolds T.B, Donovan A.J, Mikkelsen W.P, Redeker, A.G, Turril F.L, Weiner J.M. (1981)Results of a 12-year randomized trial of portacaval shunt in patients with alcoholic liver disease and bleeding varices. *Gastroenterology*, **80**: 1005–11.
3. Cello J.P, Grendell J.H, Crass R.A, Trunkey D.D, Cobb E.E, Heilbron D.C et al. (1984) Endoscopic sclerotherapy versus portocaval shunt in patients with severe cirrhosis and variceal hemorrhage. *N Eng J Med*, **311**: 1589–94.
4. Cabrera J, Maynar M, Granados R, Gorriz E, Reyes R, Rodriguez Sanromàn J.L, Mathias P, Ramirez J, Guerra C (1993) . Comunicacion intrahepàtica portosistèmica por via transyugular. Seguimiento clinico y hemodinámico. Resulata dos preliminares. *Gastroenterologia y Hepatologia*, **16**: 1–7.
5. Garcia Villareal L, Zozaya Urmeneta J.M, Quiroga Vila J, Sangro Gomez-Acebo B, Bilbao Jaureguizar J.I, Longo Areso J, Prieto valyuaena J. (1993) Prótesis intrahepàtica porto sistèmica (TIPS) en al tratamiento de la scitis refractaria. Estudio piloto. *Gastroenterologia y Hepatologia* **16**:8–12.
6. Redhead B.N, Chalmers N, Simpson K.J, Hayes P.C. (1993) Transjugular intrahepatic portasystemic stent shunting (TIPSS). A review. *J Interv Radiol*, **8**: 37–41.
7. Ring E.J, Lake J.R, Roberts J.P, Gordon, R.L, LaBerge J.M, Read A.E, Sterneck M.R, Ascher N.L. (1992) Using transjugular intrahepatic portosystemic shunt to control variceal bleeding before liver transplantation. *Ann Intern Med*, **116**: 304–309.
8. Richter G.M, Noeldge, G, Palmaz J.C, Roessle M, Slegerstetter V, Franke M, Gerok W, Wenz W, Farthman E. (1990) Transjugular intrahepatic portacaval stent shunt:preliminary clinical results. *Radiology*, **174**: 1027–1030.
9. Zemel G, Katzen B.T, Becker G.J, Benenati J.F, Sallee S. (1991) Percutaneous transjugular portosystemic shunt. *JAMA*, **266**: 390–394.
10. Sanyal A.J, Freedman A.M, Purdum pp. (1992) TIPS-associated hemolysis and encephalopathy. *Ann Intern Med*, 443–444.

